# A206 GALLSTONE IMPACTION IN THE DESCENDING COLON WITH SUCCESSFUL ENDOSCOPIC REMOVAL

**DOI:** 10.1093/jcag/gwab049.205

**Published:** 2022-02-21

**Authors:** G Malik, D Y Yang, K Wong

**Affiliations:** 1 Faculty of Medicine and Dentistry, University of Alberta, Edmonton, AB, Canada; 2 Medicine, University of Alberta, Edmonton, AB, Canada

## Abstract

**Background:**

Gallstone impaction in the colon is a rare complication of cholelithiasis, typically because of gallstones migrating into the colon via a cholecystocolonic fistula. Gastrointestinal obstruction from gallstones only develops in 0.3–0.5% of patients with cholelithiasis and accounts for 0.095% of all mechanical bowel obstruction. Successful treatment has been described with endoscopic and surgical techniques.

**Aims:**

This report aims to present a case of colonic obstruction from gallstone that was successfully treated endoscopically.

**Methods:**

We conducted a retrospective chart review on one patient including assessing the history of presenting illness along with biochemical, radiographic, and endoscopic results. We then conducted a narrative literature review on this subject matter.

**Results:**

An 85-year-old male with known cholelithiasis complicated by prior cholecystitis presented with a 2-day history of generalized abdominal pain. Computed tomography (CT) showed fistulisation between the gallbladder and hepatic flexure. Follow-up CT with rectal contrast showed lack of contrast beyond an impacted gallstone in the descending colon (Figure 1A, 1B). The patient was deemed a poor surgical candidate thus endoscopic gallstone extraction was attempted. With a pediatric colonoscope, the 41mm x 51mm gallstone was visualized 70cm from anal verge (1C). The stone was too large to be extracted en-block with snare or basket. Attempts to cut the stone at its surface with a polypectomy snare and other tools were unsuccessful because of the roundness and smoothness of the stone. Although we could chip away at the surface, we were unable to break the seal of the stone against the colon mucosa. We found the most effective method was tunneling through the stone’s centre using a closed rat-tooth forceps, then angling the tip of the scope and pulling the scope back to break off fragments from within. A polypectomy snare was used to further breakup larger fragments to prevent repeat impaction (1D). There was underlying diverticular disease and mild ischemic changes to the mucosa but no stricture. The patient did well post-procedure and was able to tolerate oral intake, pass flatus, and pass bowel movements. He was discharged 3 days later.

A recent systematic review found a total of 38 cases of gallstone obstruction of the colon. Majority of patients (73.6%) ultimately required surgical intervention. Overall mortality is 13.1%.

**Conclusions:**

Gallstone impaction in the colon is uncommon with successful endoscopic treatment seldomly described. This report describes a rare complication of gallstone disease that was successfully treated with widely available endoscopic tools.

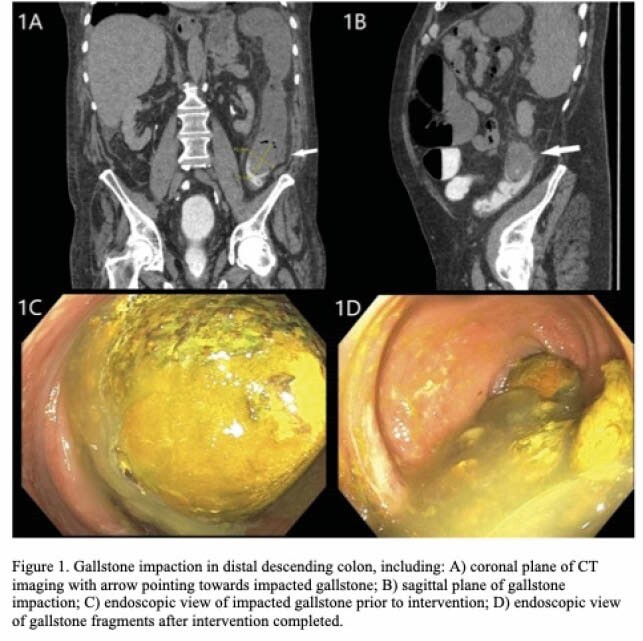

**Funding Agencies:**

None

